# A Common Assessment Space for Different Sensor Structures

**DOI:** 10.3390/s19030568

**Published:** 2019-01-29

**Authors:** Wei Wen, Ondřej Kajínek, Siamak Khatibi, Goce Chadzitaskos

**Affiliations:** 1 Department of Technology and Aesthetics, Blekinge Institute of Technology, 37179 Karlskrona, Sweden; 2 Department of Physics, Czech Technical University, 11519 Prague 1, Czech Republic; kajinond@fjfi.cvut.cz (O.K.); goce.chadzitaskos@fjfi.cvut.cz (G.C.)

**Keywords:** software-based, common space, hexagonal image, pixel arrangement, pixel form, continuous extension, resampling

## Abstract

The study of the evolution process of our visual system indicates the existence of variational spatial arrangement; from densely hexagonal in the fovea to a sparse circular structure in the peripheral retina. Today’s sensor spatial arrangement is inspired by our visual system. However, we have not come further than rigid rectangular and, on a minor scale, hexagonal sensor arrangements. Even in this situation, there is a need for directly assessing differences between the rectangular and hexagonal sensor arrangements, i.e., without the conversion of one arrangement to another. In this paper, we propose a method to create a common space for addressing any spatial arrangements and assessing the differences among them, e.g., between the rectangular and hexagonal. Such a space is created by implementing a continuous extension of discrete Weyl Group orbit function transform which extends a discrete arrangement to a continuous one. The implementation of the space is demonstrated by comparing two types of generated hexagonal images from each rectangular image with two different methods of the half-pixel shifting method and virtual hexagonal method. In the experiment, a group of ten texture images were generated with variational curviness content using ten different Perlin noise patterns, adding to an initial 2D Gaussian distribution pattern image. Then, the common space was obtained from each of the discrete images to assess the differences between the original rectangular image and its corresponding hexagonal image. The results show that the space facilitates a usage friendly tool to address an arrangement and assess the changes between different spatial arrangements by which, in the experiment, the hexagonal images show richer intensity variation, nonlinear behavior, and larger dynamic range in comparison to the rectangular images.

## 1. Introduction

The visual sensory of some of biological species can easily outperform our conventional vision technology. Inspired by such efficient machines, we have built our electronic systems which aim to capture a scenery with the same efficient style of performance by emulating the structure and function of biological counterparts. The sensor structure, sensor form, and surface shape of eye show a wide range of adaptations to meet the requirements of the organisms which bear them. Eye performance of different species vary in their visual acuity—the range of wavelengths they can detect, their sensitivity in low light, their ability to detect motion or to resolve objects, and whether they can discriminate colors [1]. The spatial sensor arrangement of the eyes plays a significant role in such variational performances [2]. The study of the evolution process of our visual system indicates how our spatial sensor arrangement is evolved and differentiated from other species and especially from the closest ones, the primates, which has resulted in the existence of variational spatial arrangement; from densely hexagonal in the fovea to a sparse circular structure in the peripheral retina. The high contrast and optimal sampling properties of our visual system are directly related to the densely hexagonal spatial arrangement.

Today’s sensor spatial arrangement is inspired by our visual system. However, we have not come further than rigid rectangular and, on a minor scale, hexagonal sensor arrangements. Some of the obstacles in developing new sensor arrangements are the difficulty in manufacturing, the cost, and rigidity of hardware components. The virtual deformation of the sensor arrangement [3] provides new possibilities for overcoming such obstacles. We need strong arguments to convince the involved partners in sensor development to implement the virtual deformation ideas. It is not enough to only show that the virtual deformation sensor arrangement is feasible, but also, that the addressing of new arrangements can be achieved easily and smoothly, without need of defining new grid structures which generally results in heavy computation. Thus, we propose a new method in the paper which eliminates the need for defining new grid structures for addressing different sensor arrangements. One direct application of the proposed method is its implementation as an assessment tool where different sensor arrangements are compared with each other; i.e., without the need for conversion of one arrangement to another one.

In this paper, we propose a method to create a common space which facilitates addressing and assessing different spatial arrangements of sensors, e.g., between the rectangular and hexagonal arrangements. Such a space is created by implementing a continuous extension of discrete Weyl Group orbit function transform which extends a discrete arrangement to a continuous one. The implementation of the space is demonstrated by comparing two types of generated hexagonal images from each rectangular image with two different methods of the half-pixel shifting and virtual hexagonal method. In the experiment, a group of ten texture images are generated with variational curviness content using ten different Perlin noise patterns, adding to an initial 2D Gaussian distribution pattern image. Then, the common space is obtained from each of the discrete images to address and assess the differences between the original rectangular image and its corresponding hexagonal image.

This paper is organized as follows. In Section 2, the addressing of arrangement is explained. Then the two types of image generation are explained in Section 3. Section 4 and Section 5 present the methodology of the common space and the experiment setup, respectively. Then the results are shown and discussed in Section 6. Finally, we summarize our work in Section 7. 

## 2. Arrangement Addressing

In relation to the assessment of two images having two different arrangements; e.g., one having square and another hexagonal arrangement, the addressing of arrangement is the most important issue by which it becomes possible to access each arrangement unit (the pixel). Such access property for any arrangement should be easy and fast in implementation, in comparison to the popular square arrangement. The problem of any arrangement, beside the square one, is manifested in finding new definitions for grid structures. Here, we elaborate on the problem for the hexagonal arrangement, which has been studied for more than four decades, and different addressing methods are suggested. A hexagonal arrangement is addressed using two oblique axes [4], also referred to as skewed coordinate system in [5], and h2 system in [6], where two basis vectors are not orthogonal. With such an oblique coordinate system, each hexagonal pixel is addressed by an ordered pair of unit vectors. A symmetrical hexagonal coordinate frame which uses three coordinates instead of two is used to represent each pixel on a grid plane [7,8]. The major advantage of this coordinate system is that there is a one-to-one mapping between hexagonal and square arrangements. Moreover, in [9], this symmetrical hexagonal coordinate frame is used to derive various affine transformations. The geometric transformations on the hexagonal grid are conveniently simplified and the symmetry property of the hexagonal grid is successfully preserved. The three-axis coordinate system is also used in [10] for mathematically handling the hexagonal arrangement. Spiral Architecture, inspired from anatomical consideration of the primate’s vision system, is proposed by [11] which is a 1D addressing system. This address grows from the center of image in powers of seven along a spiral-like curve. This addressing scheme combined with two later proposed mathematic operations, spiral addition and spiral multiplication, is the basic Spiral Architecture [11,12]. A similar single-index system for pixel addressing is proposed by modifying the Generalized Balanced Ternary system [13,14]. A virtual hexagonal structure is proposed by the authors of [15] where the hexagonal pixels do not physically exist but are recorded during image processing in the memory space. The approach demands high computation for image conversion (from one arrangement to another) for determining the locations (or the areas) of each pixel. A reduced computational complexity method is derived from the virtual hexagonal structure proposal by the authors of [16].

## 3. Image Generation

**Figure 1:**
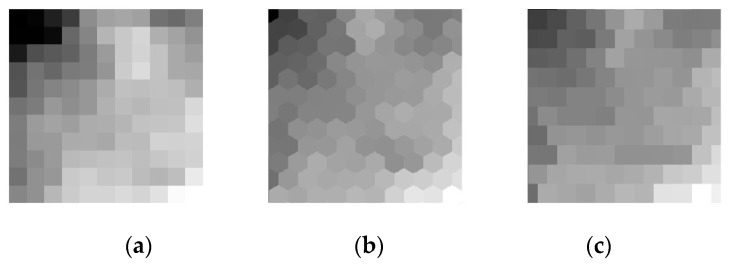
The images on three types of sensory arrangements. (**a**) The original square image (SQ); (**b**) hexagonal image (Hex_E); (**c**) half-pixel shift image (HS_E).

In this section, we explain generation of two types of images which have hexagonal arrangements. The images are generated from an original image with square arrangement. An example of such images is demonstrated in Figure 1.

### 3.1. Generation of the Virtual Hexagonal Enriched Image (Hex_E)

**Figure 2:**
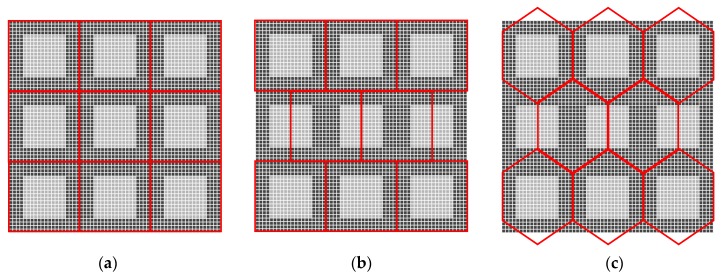
Three types of sensory arrangements. (**a**) The sensor rearrangement onto the subpixel; (**b**) the projection of the square pixels onto the hexagonal arrangement by half-pixel shifting method (i.e., HS_E image generation); (**c**) the projection of the square pixels onto the hexagonal grid in generation of hexagonal image (Hex_E).

The virtual hexagonal enriched image has a hexagonal pixel form on a hexagonal arrangement. The generation process is similar to the resampling process in [17,18], which has three steps: projecting the original image pixel intensities onto a grid of sub-pixels; estimating the values of subpixels at the resampling positions; estimating each new hexagonal pixel intensity in a new hexagonal arrangement where the subpixels are projected back to a hexagonal grid, which are shown as red grids in Figure 2. In this arrangement the distance between each two hexagonal pixels is the same and the resolution of the generated Hex_E image is the same as the original image.

### 3.2. Generation of the Virtual Half-Pixel Shift Enriched Image (HS_E)

The hexagonal grid in previous work [19,20] is mimicked by a half-pixel shift which is derived from delaying sampling by a half pixel on the horizontal direction. The red grid, which is presented in the middle of Figure 2, is the new pseudo hexagonal sampling structure whose pixel form is still square. The new pseudo hexagonal grid is derived from a usual 2D grid by shifting each even row a half pixel to the right and leaving odd rows unattached, or of course any similar translation. The virtual Half-pixel Shift Enriched image (HS_E) is generated from the original enriched image [3] which has a square arrangement.

## 4. Common Space Based on Continuous Extension

To elaborate the common space, let us start with a simple 1D example. Assuming we have a continuous 1D signal, it is not difficult to imagine that we can sample the signal with different time intervals. However, the opposite way is not so easy; i.e., to obtain the continuous signal from different time intervals. Further, this becomes even extremely difficult when we have sampled our data by a certain time interval and try to use the data to resample according to another time interval. Here, for the common space we have the last-mentioned condition where the sampled data is 2D and from the image sensor. In this relation, the choice of spatial sensor arrangement affects the sampling results as the choice of time interval in the 1D signal example. In the 2D sampling the data is sampled from a continuous surface; i.e., each spatial sensor arrangement results in certain sampling data from certain points on the continuous surface. By common space, we mean such continuous surface which is created by continuous extension of spatial data; i.e., from sampling data from certain spatial sensor arrangement a common space (a continuous surface) is generated. The common space is used to estimate the sampling data according to another spatial sensor arrangement; i.e., a common space is created by sampling data from hexagonal spatial arrangement and then the sampling data of a rectangular spatial arrangement is estimated. In this way, on the common space, we have correspondent points of each sampling point related to different spatial arrangements, which facilitates the addressing and assessing of different spatial arrangements of sensors. 

The common space is created by implementing a continuous extension of discrete Weyl Group orbit function transform. Orbit functions on the Euclidean space are symmetrized exponential functions. The symmetrization is fulfilled by a Weyl group corresponding to a Coxeter-Dynkin diagram. The values of orbit functions are repeated on copies of a fundamental domain of the affine Weyl group (determined by the initial Weyl group) in the entire Euclidean space. Recalling that the exponential functions determine the Fourier transform on Euclidean space. Correspondingly, orbit functions determine a symmetrized version of the Fourier transform which is also called an orbit function transform. One of the key properties of orbit transform is that sequence of orbit transform, and inverse orbit transform preserve the processed data. This property is preserved even when discrete orbit function in the inverse orbit transform is replaced with a continuous orbit function of the same family. In other words, for any symmetrical grid such as rectangular or hexagonal grid, in frequency domain a continuous spectrum surface can be generated from the discrete information of the grid. We call this continuous spectrum surface a common space. The creation and proof of such common space is explicated in detail in Appendix A for interested readers. 

The creation of continuous extension of the original data is independent of the data arrangement; i.e., it is possible to create common space from any spatial arrangement, such as square or hexagonal ones. We refer to these common spaces in relation to their original data arrangements, such as CSE_sq or CSE_hex for the created common spaces from square and hexagonal arrangement, respectively.

On the common space, any grid structure is applied virtually; i.e., the corresponding addressing of each pixel position from different arrangements are done on the common space. Thus, by knowing the pixel form of each arrangement, the intensity value of each corresponding pixel is determined at the pixel position on the common space. 

## 5. Experimental Setup

Evaluating the proposed common space method in assessing different sensor structures is based on using different generated images. In Section 3, the generated procedures of those types of image, which are used in the evaluation, are all types of image that are originated from a rectangular arrangement. Thus, generating images based on rectangular arrangement is essential for experimental evaluation. On the other hand, to evaluate the addressing accuracy of the common space usage, we need to generate such images which also have a content with random spatial variation in each pixel. This is because by using the common space only one coordinate system is used to address each pixel position and obtain its intensity value in two different arrangements; i.e., each pixel position and intensity value of the originate arrangement to the common space is known, but the correspondent position and intensity value on the other arrangement is estimated using the common space surface. In relation to this, the evaluation of addressing accuracy can be achieved by measuring the estimations error. The statistical validation of the estimations error requires the random spatial variation in each pixel; i.e., as spatial variation in natural images. The estimations error can be measured for all pixels of each two experimental images, using the common space addressing, or selected amount of their correspondent pixels. In the experiments we used the latter option. To ensure that the selected pixels represent different intensity levels it requires to generate the experimental images with a certain intensity model; e.g., a Gaussian model. 

**Figure 3:**
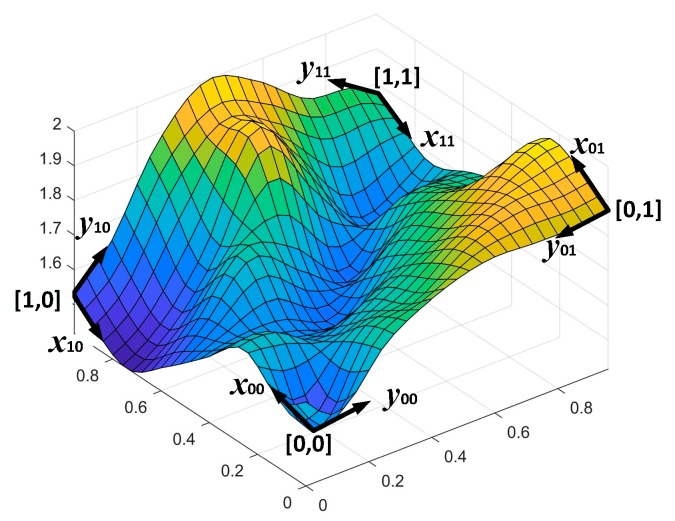
At integer grid points, 2D Perlin noise interpolates and smooths between pseudorandom gradients.

**Figure 4:**
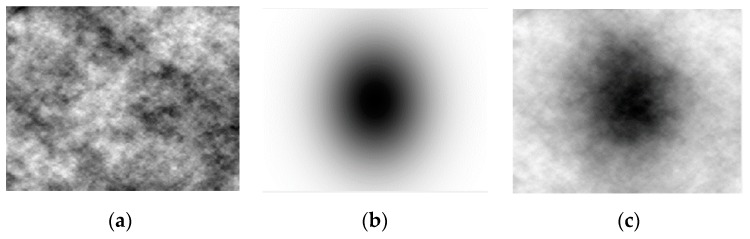
Generation of an SQ image (**a**) is added to a Gaussian image: PI; (**b**): GI; (**c**): a random Perlin noise image.

An image dataset is created which consists of 10 high resolution (4096 by 2160) original images (SQs) and their converted ones, of type of HS_E and Hex_E images with the same resolution; i.e., the dataset has a total of 30 images, where the interval of subpixel is 30. The conversion process is elaborated on in Section 3. Each of the ten original images is generated by adding a Gaussian image (GI) to a random Perlin noise image (PI). The *GI* contributes to obtain all possible tonal levels in range of 0–255 gray levels in each original image. Each *GI* is generated by

(1) $$GI=255*e^{-\left(\frac{x^{2}}{2\sigma_{1}^{2}}+\frac{y^{2}}{2\sigma_{2}^{2}}\right)}$$

where $\sigma_{1}$ and $\sigma_{2}$ are 1920 and 1280 respectively and the original images *SQs* is obtained by:

(2) $$SQ_{j}=GI+PI_{j}$$

where j is the image index number. The values of $\sigma_{1}$ and $\sigma_{2}$ are approximately half of the image resolution in each direction. Based on the rule of thumb, *GI* represents fully a Gaussian intensity model where the values of $\sigma_{1}$ and $\sigma_{2}$ are one third of image resolution in each direction. In this relation *GI* is not fully a representative of a Gaussian intensity model. This is to prevent obtaining significantly lower level intensity values which can affect evaluation of addressing accuracy. By generating the PI image, a pseudo-random spatial variation in each pixel is obtained which simulates variational curviness content; i.e., we imitate the appearance of textures in natural images by a controlled random process. In this way, using *GI* and *PI*, each original image of the dataset is generated to have natural images properties and with wider range of variation than exists in a captured natural image. Each *PI* is generated by implementing the Perlin noise algorithm [21,22] where each pixel of the image; *PI*(x, y), is computed by two major steps: (a) projection of pixel vector position on pseudorandom gradients of ${\vec{g}}_{00}=\left[x_{00},y_{00}\right]$, ${\vec{g}}_{01}=\left[x_{01},y_{01}\right]$, ${\vec{g}}_{10}=\left[x_{10},y_{10}\right]$, and ${\vec{g}}_{11}=\left[x_{11},y_{11}\right]$ at integer points [0,0], [0,1], [1,0], and [1,1], respectively, (b) interpolation and smoothing between points ‘value at the integer points by a cubic spline function $S\left(x\right)=x^{2}\left(3-2x\right)$ and a linear interpolation function L(ε,x,y)=x+ε(y−x) as shown in Figure 3 and explained by algorithm steps in Table 1. The PI contributes to obtain all possible tonal levels in range of 0–255 gray levels. The range of $SQ_{j}$ images; a combination of GI and PI images where each has a range of 0–255 tonal levels, are normalized to obtain images with range of 0–255 tonal levels. The generation of SQ images is demonstrated in Figure 4.

## 6. Results and Analysis 

**Figure 5:**
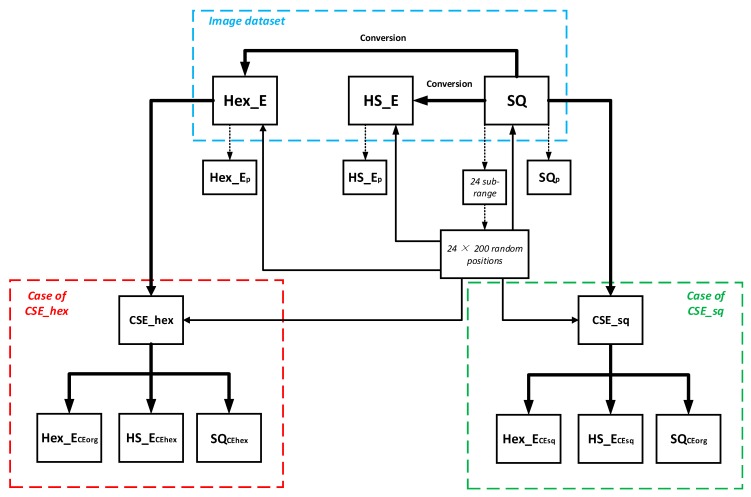
The flowchart to discuss and analyze the obtained results.

In this section, we show the addressing and assessment feasibility of three types of images of SQ, HS_E, and Hex_E (i.e., having different pixel arrangements) using the common space. There are ten of such triple types of images in the dataset and for each triple image type the results were obtained in three stages of general preparation, case of CSE_sq and case of CSE_hex as it is shown in the flowchart of Figure 5. The blue, green and red dash-line squares represent the image dataset generation, case of CSE_sq and case of CSE_hex respectively. The dot arrow shows the pixels are selected in the Hex_E, HS_E and SQ images. The thick and thin arrows represent the process of image generation and applying the selected pixels on the images respectively. Table 2 lists the symbols in Figure 5 with their meanings. We explain the three stages and then discuss and analyze the obtained results which indicate the feasibility and accuracy of addressing and assessment of random pixels from one arrangement to another one. 

### 6.1. General Preparation

**Figure 6:**
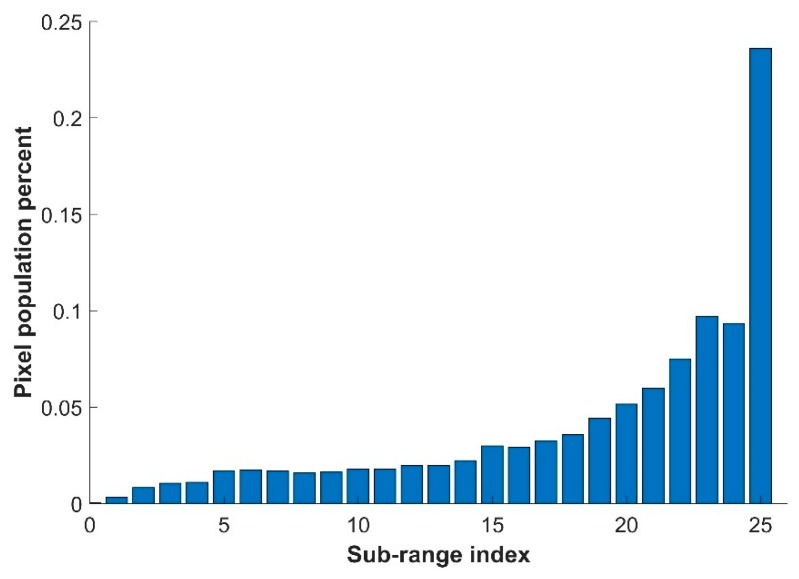
A typical pixel population for 25 intensity sub-ranges.

Each SQ image in the data set is an eight bits image; i.e., the range of intensity values is between 0 and 255. The pixels of each SQ image are partitioned by having 24 intensity sub-ranges (e.g., 10–19, …, 190–199, 240–250) to investigate in more detail the tonal variation. In each sub-range, 200-pixel positions are selected randomly in each SQ image; i.e., 24 by 200 pixels are chosen randomly meanwhile assuring to have different tonal levels and representative of the whole intensity range. The 24 intensity sub-ranges are related to the statistical requirement of having a pixel population in which we can select 200 pixels positions. According to our observation from the generated images, a binning of 10 tonal levels could fulfill the requirement where each intensity sub-range has at least a pixel population of 1%. Figure 6 shows a typical pixel population for 25 intensity sub-ranges. The first intensity sub-range; with tonal levels between 0–9. And the last sub-range with tonal levels between 251–255 have less than the pixel population of 1% which accordingly will be discard in the pixel selection process. The 200 random pixels in each intensity sub-range is because they contain sufficient spatial intensity variation information in a certain sub-range of tonal variations to underpin statistical analysis. Using the pixel positions, the relative intensity values from SQ, HS_E and Hex_E images are organized in new images of $SQ_{p}$, $HS\_E_{p}$, and $Hex\_E_{p}$ respectively; each with size of 200 by 24. The pixels of each column of such an image are ordered by sorting the linear indexing of the 200 random selected pixels in each intensity-subrange.

### 6.2. In Case of CSE_sq 

The common space of each SQ image, CSE_sq, is created according to Section 4. Using the common space of CSE_sq and the pixel positions of a SQ image the corresponding pixel positions and the related intensity values are estimated for SQ, HS_E, and Hex_E image types. Accordingly, in correspondent to a $SQ_{p}$, three images of $SQ_{CEorg},HS\_E_{CEsq},\;and\;Hex\_E_{CEsq}$ are generated.

### 6.3. In Case of CSE_hex 

The common space of each Hex_E image, CSE_hex, is created according to Section 4. As with Case 6.1, by using the pixel positions of SQ image and the common space of CSE_hex, the corresponding pixel positions and the related intensity values are estimated for SQ, HS_E, and Hex_E image types. Accordingly, corresponding to a $Hex\_E_{p}$ three images of $SQ_{CEhex}$, $HS_{CEhex}$ and $Hex_{CEorg}$ are generated. 

### 6.4. Analysis of the Two Cases

In cases of CSE_sq or CE_hex, the images with a square or a hexagonal arrangement originate the respective common spaces. Generally, in the process of obtaining the results by using a common space and a pixel position in the originated image to the common space, the corresponding pixel position and its intensity value are estimated for another type of image which has another arrangement in comparison to the originated image. Here, we address the three questions of (a) How different are any two generated common spaces which are originated from two different arrangements; e.g., the comparison of generated $SQ_{CEorg}$ (representative of CSE_sq common space) and $Hex\_E_{CEorg}$ (representative of CSE_hex common space)? (b) How similar are any generated common space and its originated image; e.g., the comparison of $SQ_{p}$ to $SQ_{CEorg}$ or $Hex\_E_{p}$ to $Hex\_E_{CEorg}$? (c) What is the accuracy of implementing any common space in addressing and assessment between two types of arrangements; e.g., from SQ to Hex_E?

**Figure 7:**
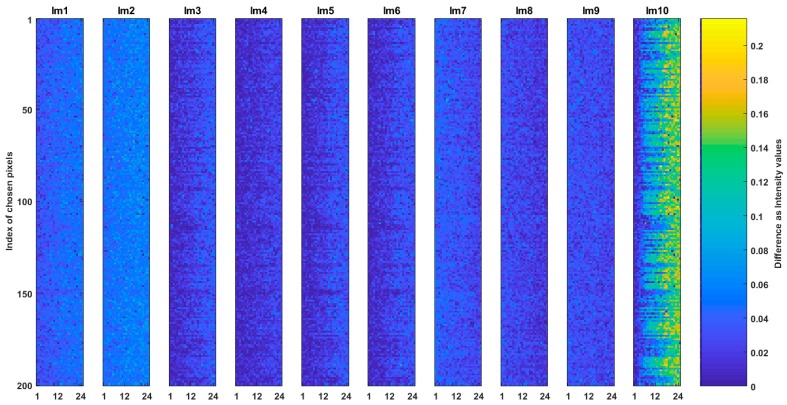
Comparison of CSE_sq and CSE_Hex. The absolute intensity value difference of ten $SQ_{CEorg}$ and $Hex\_E_{CEorg}$ are shown.

We generated ten CSE_sq and ten CSE_hex common spaces from the related images in the dataset; i.e., each SQ image and its converted Hex_E image were used to create each related CSE_sq and CSE_hex (a pair of common spaces). For each pair of the common spaces a pixel set of 200 chosen pixels (see Section 6.1 and Section 6.2) of the originated images were chosen and organized as images. In this way, ten $SQ_{CEorg}$ and ten $Hex\_E_{CEorg}$ images are obtained where each has size of 200 by 24 and represent the relative common space. Question (a) is answered by comparison of the $SQ_{CEorg}$ and $Hex\_E_{CEorg}$ images. Figure 7 shows the results of such comparisons where the absolute intensity value difference of ten $SQ_{CEorg}$ and $Hex\_E_{CEorg}$ are measured. In the figure the colors from blue to yellow indicate that the difference value increases from 0 to 0.2. The total mean square error (MSE) between images shown in Figure 7 is 0.002 and multiple correlation among the images is 99.39%. The low MSE and high correlation indicate that it is feasible to create almost the same common space for the two arrangements of square and hexagonal. The created common spaces are close, but as expected, is not exactly the same; e.g., a hexagonal arrangement has richer frequency spectrum than the square one which contributes to obtain richer frequency spectrum on respective common space [23].

**Figure 8:**
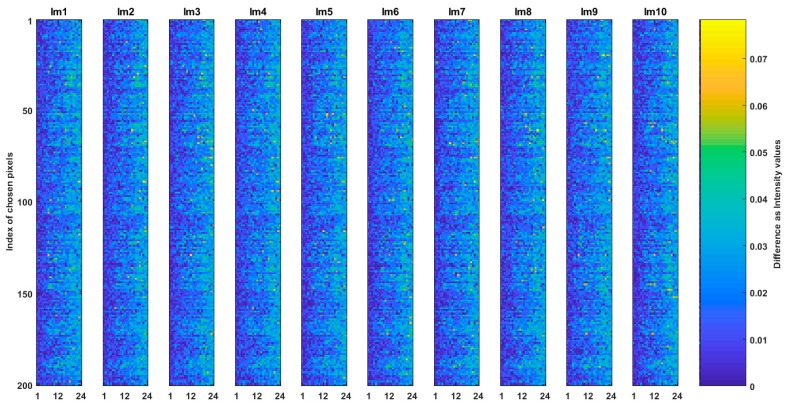
Comparison of the common space of CSE_sq and its originated image of SQ. The absolute intensity value difference of ten $SQ_{p}$ and $SQ_{CEorg}$ are shown.

**Figure 9:**
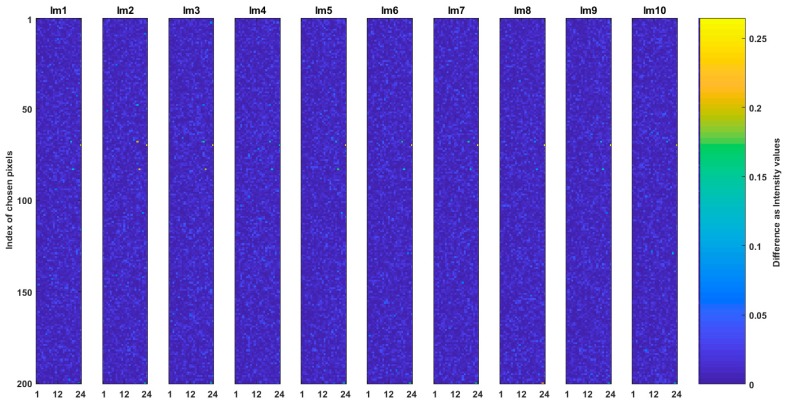
Comparison of the common space of CSE_hex and its originated image of Hex_E. The absolute intensity value difference of ten $Hex\_E_{p}$ and $Hex\_E_{CEorg}$ are shown.

Question (b) is answered by the comparison of $SQ_{p}$ to $SQ_{CEorg}$ and $Hex\_E_{p}$ to $Hex\_E_{CEorg}$ images. The results of such comparisons where the absolute intensity value difference of ten of $SQ_{p}$ to $SQ_{CEorg}$ and $Hex\_E_{p}$ to $Hex\_E_{CEorg}$ images are shown in Figure 8 and Figure 9 respectively. The total MSE between and multiple correlation among the images in Figure 8 is 0.0005 and 99.93% respectively. In Figure 9, the total MSE between images is 0.00019 and multiple correlation among them is 99.85%. The low MSE and high correlation in the results of the figures indicate that the generated common spaces are very alike to their respective originated images but they are not strictly the same.

**Figure 10:**
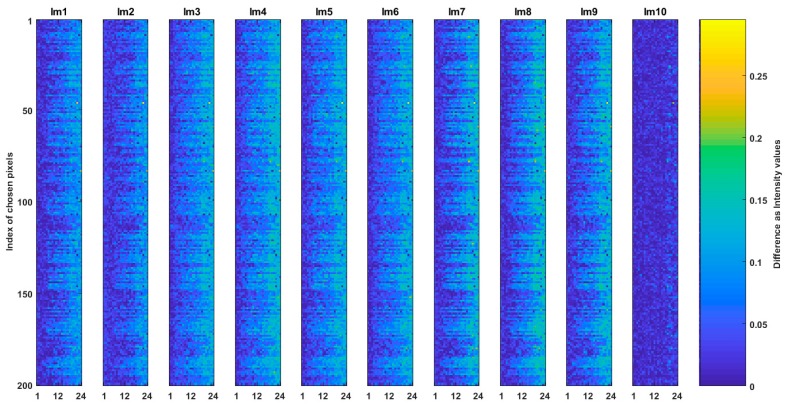
Comparison of ten $SQ_{CEhex}$ and $SQ_{p}$ images.

**Figure 11:**
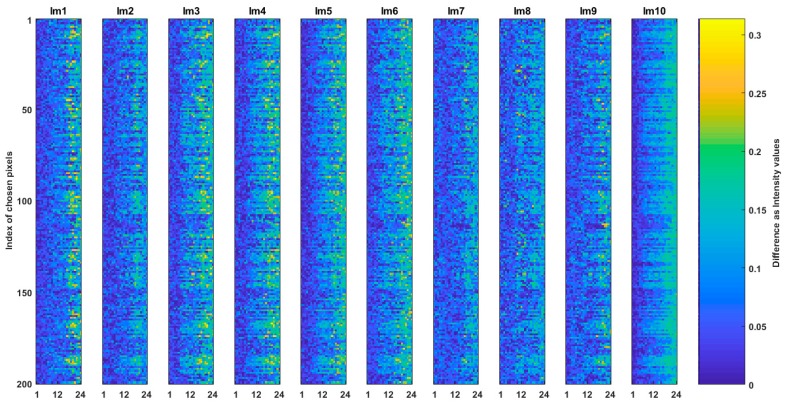
Comparison of $Hex_{CEsq}$ and $Hex\_E_{p}$.

Question (c) is answered by examining each case of CSE_sq and CSE_hex in addressing and assessment between different types of arrangements. In case of CSE_sq ten of each $Hex\_E_{CEsq}$, $HS\_E_{CEsq}$, and $SQ_{CEorg}$ images are obtained, and they are compared to $Hex\_E_{p}$, $HS\_E_{p}$, and $SQ_{p}$ (i.e., the representatives of the images of Hex_E, Hs_E, and SQ). In case of CSE_hex ten of each $Hex\_E_{CEorg}$, $HS\_E_{CEhex}$, and $SQ_{CEhex}$ images are obtained, and they are compared to $Hex\_E_{p}$, $HS\_E_{p}$, and $SQ_{p}$. Figure 10 and Figure 11 show two examples of such comparison between $SQ_{CEhex}$ to $SQ_{p}$ and $Hex_{CEsq}$ to $Hex\_E_{p}$ respectively.

In Figure 10, the total MSE between the ten $SQ_{CEhex}$ and $SQ_{p}$ is 0.0059 and multiple correlation between them is 99.03%. In Figure 10 the total MSE between the ten of $Hex_{CEsq}$ and $Hex\_E_{p}$ is 0.0099 and correlation between the pixel sets is 98.26%. The results in the Figure 7, Figure 8, Figure 9 and Figure 10 show that by implementing the common space, it is feasible to address different arrangements where the intensity difference between any random pixel which is addressed via common space or via conversion is very small.

**Figure 12:**
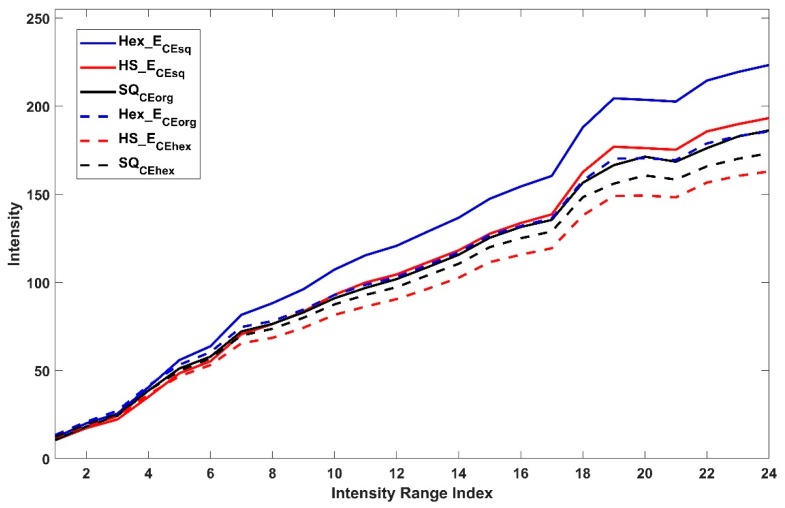
Intensity average of ten corresponding pixel sets of each $Hex\_E_{CEsq}$, $HS\_E_{CEsq}$, $SQ_{CEorg}$ or $Hex\_E_{CEorg}$, $HS\_E_{CEhex}$, $SQ_{CEhex}$.

**Figure 13:**
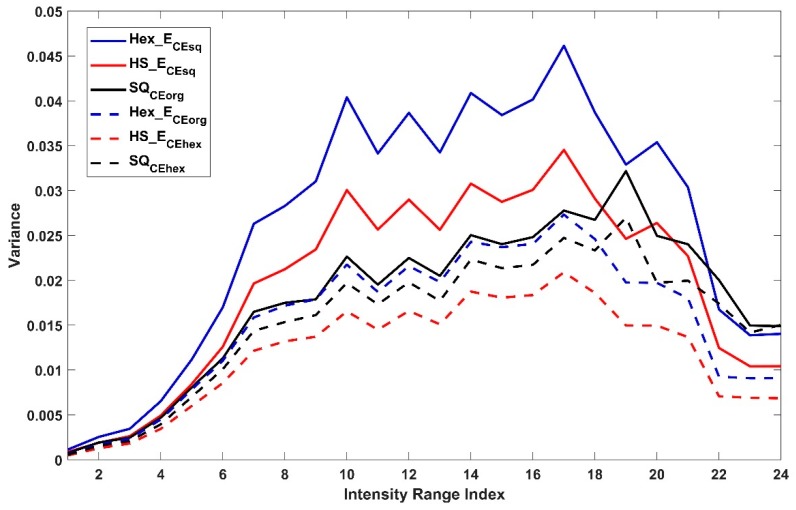
Variance of ten corresponding pixel sets of each $Hex\_E_{CEsq}$, $HS\_E_{CEsq}$, $SQ_{CEorg}$ or $Hex\_E_{CEorg}$, $HS\_E_{CEhex}$, $SQ_{CEhex}$.

**Figure 14:**
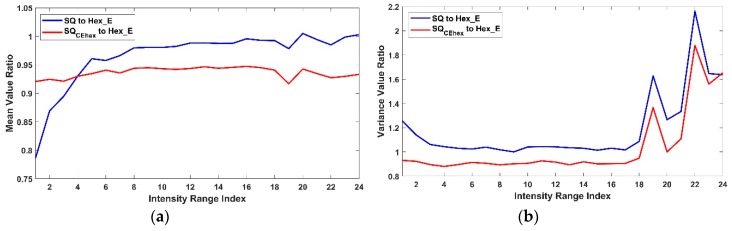
The mean (**a**) and variance (**b**) of ratio values of ten corresponding pixel sets between each SQ and $SQ_{CEhex}$ to Hex_E.

In each case of CSE_sq or CSE_hex, the intensity average and variance in the 24 tonal sub-ranges of ten corresponding pixel sets of each $Hex\_E_{CEsq}$, $HS\_E_{CEsq}$, $SQ_{CEorg}$ or $Hex\_E_{CEorg}$, $HS\_E_{CEhex}$, $SQ_{CEhex}$ are shown in Figure 12 and Figure 13 respectively. The figures show that it is feasible to assess pixels on different arrangements due to the estimation of pixel position and the intensity value in different arrangement by using common space and without the need for any conversion means (see Section 4). The pixel sets from hexagonal arrangement show the highest average intensity value and variance in each type of common space indicating richer intensity variation and larger dynamic range compared to SQ the other pixel sets. Figure 14 shows the mean (a) and variance (b) of ratio values of ten corresponding pixel sets between each SQ and $SQ_{CEhex}$ to Hex_E image. The mean (a) shows the nonlinear relation between SQ to Hex_E which was previously shown in [3,18]. The mean (a) also shows that the relation between $SQ_{CEhex}$ to Hex_E is similar to the relation between SQ to Hex_E and behaves in a nonlinear manner. The variance (b) shows that the relation between SQ and $SQ_{CEhex}$ to Hex_E are similar and nonlinear.

The pixel sets on corresponding arrangements via two types of common spaces are compared and shown in Table 3. The comparison shows the correlation and MSE relation between each pair of pixel sets. The results in the table indicate the feasibility of addressing each type of common space to the same type of arrangement due to small MSE and high correlation values. The similar results of correlation and MSE in Table 4 shows the assessment feasibility of different arrangements by comparison of the pixel sets on different arrangement and via two types of common spaces.

## 7. Conclusions

In the paper we proposed a method to create a common space, which eliminates the need for defining new grid structures for addressing different sensor arrangements. We showed the feasibility of addressing and assessing different spatial arrangements of sensors, specifically between the rectangular and hexagonal arrangements. We explained how the common space is created by implementing a continuous extension of discrete Weyl Group orbit function transform, which extends a discrete arrangement to a continuous one. The results indicate that the common space facilitates an easy tool for addressing any pixel position on any arrangement and specifically we showed such facilitation on square and hexagonal arrangements. It was also shown that the tool has significant property to assess the changes between different spatial arrangements by which, in the experiment, the pixel sets on hexagonal images show richer intensity variation, nonlinear behavior, and larger dynamic range in comparison to the pixel sets on rectangular images. 

## Figures and Tables

**Table 1:** The algorithm of implemented 2D Perlin noise.

0.	Input $\vec{P}=\left[x,y\right]$
1.	$S_{x}=S\left(x\right)$
2.	$S_{y}=S\left(y\right)$
3.	$u_{a}=\vec{P}\cdot {\vec{g}}_{00}$
4.	$v_{a}=\vec{P}\cdot {\vec{g}}_{10}$
5.	$a=L(S_{x},u_{a},v_{a}$)
6.	$u_{b}=\vec{P}\cdot {\vec{g}}_{01}$
7.	$v_{b}=\vec{P}\cdot {\vec{g}}_{11}$
8.	$b=L(S_{x},u_{b},v_{b}$)
9.	Output $L(S_{y},a,b$)

**Table 2:** Description of used symbols.

Symbol	Full Name and Size	Sensor Arrangement	Originated from	Method
SQ	Square image 4096 × 2160	square	-	-
Hex_E	Hexagonal enriched image 4096 × 2160	hexagonal	SQ	Conversion
HS_E	Half-pixel shift image 4096 × 2160	square	SQ	Conversion
$SQ_{p}$	Square matrix image 200 × 24	square	SQ	Pixel selection on SQ
$\mathrm{Hex}\_E_{p}$	Hexagonal enriched matrix image 200 × 24	hexagonal	Hex_E	Pixel selection on Hex_E
$\mathrm{HS}\_E_{p}$	Half-pixel shift matrix image 200 × 24	square	HS_E image	Pixel selection on HS_E
CSE_sq	Common Space surface	continuous extension	SQ image	New method, see Section 4
CSE_hex	Common Space surface	continuous extension	Hex_E image	New method, see Section 4
$SQ_{\mathrm{CEorg}}$	Estimated Square matrix image 200 × 24	square	CSE_sq	Pixel selection on the CSE_sq
$\mathrm{Hex}\_E_{\mathrm{CEsq}}$	Estimated Hexagonal matrix image 200 × 24	hexagonal	CSE_sq	Pixel selection on the CSE_sq
$\mathrm{HS}\_E_{\mathrm{CEsq}}$	Estimated Half-pixel shift matrix image 200 × 24	square	CSE_sq	Pixel selection on the CSE_sq
$SQ_{\mathrm{CEhex}}$	Estimated Square matrix image 200 × 24	square	CSE_hex	Pixel selection on the CSE_hex
$\mathrm{He}x_{\mathrm{CEorg}}$	Estimated Hexagonal matrix image 200 × 24	hexagonal	CSE_hex	Pixel selection on the CSE_hex
$HS_{\mathrm{CEhex}}$	Estimated Half-pixel shift matrix image 200 × 24	square	CSE_hex	Pixel selection on the CSE_hex

**Table 3:** Comparison of pixel sets on corresponding arrangements via two types of common spaces.

		Image Index									
	Pair of Pixel Sets	No.1	No.2	No.3	No.4	No.5	No.6	No.7	No.8	No.9	No.10
**Corre-lation**	$SQ_{\mathrm{CEorg}}$ $SQ_{\mathrm{CEhex}}$	99.63%	99.63%	99.62%	99.61%	99.66%	99.64%	99.63%	99.63%	99.61%	99.63%
	$\mathrm{HS}\_E_{\mathrm{CEsq}}$ $\mathrm{HS}\_E_{\mathrm{CEhex}}$	98.25%	98.45%	98.28%	98.39%	98.79%	98.32%	98.41%	97.76%	98.49%	99.79%
	$\mathrm{Hex}\_E_{\mathrm{CEsq}}$ $\mathrm{Hex}\_E_{\mathrm{CEorg}}$	98.23%	98.44%	98.26%	98.39%	98.78%	98.32%	98.41%	97.77%	98.49%	99.78%
**MSE**	$SQ_{\mathrm{CEorg}}$ $SQ_{\mathrm{CEhex}}$	0.0024	0.0021	0.0038	0.0046	0.0040	0.0038	0.0038	0.0044	0.0039	0.0005
	$\mathrm{HS}\_E_{\mathrm{CEsq}}$ $\mathrm{HS}\_E_{\mathrm{CEhex}}$	0.0070	0.0054	0.0092	0.0085	0.0080	0.0091	0.0055	0.0065	0.0060	0.0080
	$\mathrm{Hex}\_E_{\mathrm{CEsq}}$ $\mathrm{Hex}\_E_{\mathrm{CEorg}}$	0.0104	0.0080	0.0136	0.0125	0.0119	0.0135	0.0081	0.0095	0.0088	0.01193

**Table 4:** Assessment by comparison of the pixel sets on different arrangements via two types of common spaces.

		Image Index									
	Pair of Pixel Sets	No.1	No.2	No.3	No.4	No.5	No.6	No.7	No.8	No.9	No.10
**Corre-lation**	$SQ_{\mathrm{CEorg}}$ $\mathrm{HS}\_E_{\mathrm{CEhex}}$	98.19%	98.17%	98.00%	98.00%	97.96%	97.89%	98.02%	97.96%	97.99%	98.07%
	$SQ_{\mathrm{CEorg}}$ $\mathrm{Hex}\_E_{\mathrm{CEhex}}$	98.23%	98.16%	98.01%	97.94%	97.95%	97.88%	98.04%	97.98%	97.97%	98.07%
	$\mathrm{HS}\_E_{\mathrm{CEsq}}$ $SQ_{\mathrm{CEhex}}$	95.99%	96.19%	96.12%	96.20%	96.67%	96.16%	96.03%	95.49%	96.08%	97.76%
	$\mathrm{HS}\_E_{\mathrm{CEsq}}$ $\mathrm{Hex}\_E_{\mathrm{CEorg}}$	98.22%	98.42%	98.22%	98.37%	98.77%	98.28%	98.39%	97.72%	98.47%	99.75%
	$\mathrm{Hex}\_E_{\mathrm{CEsq}}$ $SQ_{\mathrm{CEhex}}$	95.98%	96.19%	96.13%	96.20%	96.66%	96.18%	96.02%	95.52%	96.07%	97.76%
	$\mathrm{Hex}\_E_{\mathrm{CEsq}}$ $\mathrm{HS}\_E_{\mathrm{CEhex}}$	98.24%	98.46%	98.30%	98.40%	98.79%	98.35%	98.41%	97.79%	98.49%	99.79%
	$SQ_{\mathrm{CEorg}}$ $\mathrm{HS}\_E_{\mathrm{CEsq}}$	96.43%	96.78%	96.55%	96.51%	96.93%	96.59%	96.55%	96.20%	96.51%	98.05%
	$SQ_{\mathrm{CEorg}}$ $\mathrm{Hex}\_E_{\mathrm{CEsq}}$	96.42%	96.78%	96.56%	96.51%	96.91%	96.59%	96.54%	96.22%	96.50%	98.04%
	$SQ_{\mathrm{CEhex}}$ $\mathrm{HS}\_E_{\mathrm{CEhex}}$	97.93%	97.84%	97.88%	97.86%	97.87%	97.77%	97.68%	97.70%	97.70%	97.99%
	$SQ_{\mathrm{CEhex}}$ $\mathrm{Hex}\_E_{\mathrm{CEorg}}$	97.98%	97.84%	97.90%	97.80%	97.86%	97.76%	97.72%	97.72%	97.68%	97.99%
	$\mathrm{HS}\_E_{\mathrm{CEsq}}$ $\mathrm{Hex}\_E_{\mathrm{CEorg}}$	99.98%	99.98%	99.98%	99.98%	99.98%	99.98%	99.98%	99.98%	99.98%	99.98%
	$HS_{\_\mathrm{ECEhex}}$ $\mathrm{Hex}\_E_{\mathrm{CEorg}}$	99.90%	99.90%	99.89%	99.90%	99.90%	99.89%	99.90%	99.90%	99.89%	99.91%
**MSE**	$SQ_{\mathrm{CEorg}}$ $\mathrm{HS}\_E_{\mathrm{CEhex}}$	0.0044	0.0037	0.0072	0.0071	0.0075	0.0068	0.0039	0.0047	0.0043	0.0069
	$SQ_{\mathrm{CEorg}}$ $\mathrm{Hex}\_E_{\mathrm{CEorg}}$	0.9823	0.9816	0.9801	0.9794	0.9795	0.9788	0.9804	0.9798	0.9797	0.9807
	$\mathrm{HS}\_E_{\mathrm{CEsq}}$ $SQ_{\mathrm{CEhex}}$	0.0089	0.0080	0.0096	0.0100	0.0086	0.0100	0.0100	0.0107	0.0099	0.0032
	$\mathrm{HS}\_E_{\mathrm{CEsq}}$ $\mathrm{He}x_{\_\mathrm{ECEorg}}$	0.0031	0.0027	0.0032	0.0029	0.0022	0.0031	0.0034	0.0036	0.0034	0.0012
	$\mathrm{Hex}\_E_{\mathrm{CEsq}}$ $SQ_{\mathrm{CEhex}}$	0.0255	0.0236	0.0274	0.0283	0.0259	0.0283	0.0278	0.0288	0.0274	0.0120
	$\mathrm{Hex}\_E_{\mathrm{CEsq}}$ $\mathrm{HS}\_E_{\mathrm{CEhex}}$	0.0238	0.0204	0.0288	0.0274	0.0270	0.0287	0.0201	0.0220	0.0212	0.0279
	$SQ_{\mathrm{CEorg}}$ $\mathrm{HS}\_E_{\mathrm{CEsq}}$	0.0051	0.0048	0.0049	0.0050	0.0043	0.0049	0.0048	0.0052	0.0050	0.0030
	$SQ_{\mathrm{CEorg}}$ $\mathrm{Hex}\_E_{\mathrm{CEsq}}$	0.0148	0.0148	0.0136	0.0131	0.0123	0.0141	0.0138	0.0137	0.0135	0.01257
	$SQ_{\mathrm{CEhex}}$ $\mathrm{HS}\_E_{\mathrm{CEhex}}$	0.0018	0.0020	0.0021	0.0019	0.0022	0.0021	0.0023	0.0023	0.0021	0.0078
	$SQ_{\mathrm{CEhex}}$ $\mathrm{Hex}\_E_{\mathrm{CEhex}}$	0.0053	0.0064	0.0042	0.0051	0.0044	0.0046	0.0089	0.0083	0.0079	0.0029
	$\mathrm{HS}\_E_{\mathrm{CEsq}}$ $\mathrm{Hex}\_E_{\mathrm{CEsq}}$	0.0061	0.0061	0.0061	0.0061	0.0061	0.0061	0.0061	0.0061	0.0061	0.0061
	$\mathrm{HS}\_E_{\mathrm{CEhex}}$ $\mathrm{Hex}\_E_{\mathrm{CEorg}}$	0.0039	0.0040	0.0036	0.0036	0.0036	0.0036	0.0041	0.0040	0.0041	0.0035

## Appendix A

### A.1. Root System

A root system is a configuration of vectors in a Euclidean space satisfying certain geometrical properties. Let us define a root system as a finite set of non-trivial vectors $∆=\left\{\alpha_{i}\in R^{n}\right\}$ that fulfil three conditions:

- Roots $\alpha_{i}\in ∆\mathrm{span}R^{n}$
- If $\alpha_{i}\in ∆$, then $\lambda \alpha \in ∆\overset{}{\Leftrightarrow }\lambda \in \left\{-1,1\right\}$: every root system contains only two scalar multiples of each root: the root itself and its reflection,
- $\alpha ,\beta \in ∆\Rightarrow \gamma_{\alpha }\beta \in ∆$: root system is closed under reflection with respect to hyperplanes orthogonal to roots. $\gamma_{\alpha }\beta$ denotes reflection of root β with respect to hyperplane orthogonal to root α.

So-called crystallographic root systems also fulfil the fourth condition: $\forall \alpha ,\beta \in ∆:\frac{2\left(\alpha ,\beta \right)}{\left(\alpha ,\alpha \right)}\in Z$

We can unambiguously choose a set of simple roots Σ⊂Δ. Simple roots fulfil two extra conditions:

- all simple roots are linearly independent,
- every root $\alpha_{i}\in ∆$, can be expressed as a linear combination of simple roots, such that all coefficients of this linear combinations are either all non-negative (such root is called positive root), or are all non-positive (negative root).

When each root is expressed as a linear combination of simple roots, we can introduce ordering of roots. So-called highest of roots is denoted ξ and is expressed as $\xi =m_{1}\alpha_{1}+m_{2}\alpha_{2}$, where $\alpha_{1},\;\alpha_{2}$ are simple roots. Coefficients $m_{1},\;m_{2}$ are called marks. There are several significant sets of vectors that are related to each root system: set of co-roots $\left(a_{i}^{\vee }\right)$, weights $\left(\omega_{i}\right)$ and co-weights $\left(\omega_{j}^{\vee }\right)$. Co-roots and co-weights are normalized variants of roots and weights, respectively:

(A1) $$a_{i}^{\vee }=\frac{2\alpha_{i}}{\langle \alpha_{i},\alpha_{i}\rangle }$$

(A2) $$\omega_{j}^{\vee }=\frac{2\omega_{i}}{\langle \omega_{i},\omega_{i}\rangle }$$

Roots and weights are dual to each other, in the following sense:

(A3) $$\langle \alpha_{j},\omega_{j}^{\vee }\rangle =\langle a_{i}^{\vee },\omega_{i}\rangle =\delta_{\mathrm{ij}}$$

These four sets of vectors are used to form four lattices (root lattice Q, co-root lattice $Q^{\vee }$ weight lattice P and co-weight lattice $P^{\vee }$) which will be used in the definition of discrete orbit function. All four lattices are defined in the following manner:

$$\begin{array}{c} Q={Z\alpha }_{1}+{Z\alpha }_{2}\;Q^{\vee }={Z\alpha }_{1}^{\vee }+{Z\alpha }_{2}^{\vee }\\ P={Z\omega }_{1}+{Z\omega }_{2}\;P^{\vee }={Z\omega }_{1}^{\vee }+{Z\omega }_{2}^{\vee } \end{array}$$

Each of these lattices can have its non-negative part (denoted with superscript +) and positive part (denoted with superscript ++).

### A.2. Weyl Groups

When having root system ∆ composed of roots $\alpha_{i}$, we define $r_{i}$, as a reflection with respect to root $\alpha_{i}$. Set of reflections $r_{i}$ will generate so-called Weyl group W. Affine Weyl group is an extension of Weyl group, it is generated by reflections $r_{i}$ plus reflection $r_{0}$, which is a reflection with respect to highest root ξ Weyl group orbit of point x is a finite set of points generated by all actions of Weyl group W. Similarly, the affine orbit of point x is generated by all actions of $W^{\mathrm{aff}}$ on point x, however, affine orbit is an infinite set, due to the reflection $r_{0}$. Fundamental region of $W^{\mathrm{aff}}$ is a closed subset of $R^{n}$ such that it contains exactly one point of each affine Weyl group orbit. The fundamental region for affine Weyl groups in R^2^ space can be chosen a convex hull of points $\left\{0,\frac{\omega_{1}^{\vee }}{m_{1}},\frac{\omega_{2}^{\vee }}{m_{2}}\right\}$.

The dual root system $\Delta^{\vee }$ is obtained as system of co-roots. Reflections related to dual root system $\Delta^{\vee }$ generate dual Weyl group $\hat{W}$. Dual Weyl group $\hat{W}$ has its fundamental region $F^{\vee }$ and can be extended to affine dual Weyl group $\hat{W^{\mathrm{aff}}}$. Since roots and co-roots differ only with their lengths, both W and $\hat{W}$ generated by the same sets of reflections. However, highest co-root $\eta =m_{1}^{\vee }\alpha_{1}^{\vee }+m_{2}^{\vee }\alpha_{2}^{\vee }$ differs from highest root ξ in both length and direction, and thus the dual affine Weyl group $\hat{W^{\mathrm{aff}}}$ is not the same as $W^{\mathrm{aff}}$. As a consequence, $F\neq F^{\vee }$. Root systems are not the only way how to generate Weyl groups. Roots of simple Lie algebras coincide with simple roots-designation of Lie algebras are often used to designate Weyl groups generated by reflections with respect to roots of given Lie algebra.

### A.3. Orbit Functions and Orbit Transforms

Weyl group orbit functions were defined for all simple Lie algebras $\left(A_{n},\;B_{n},\;C_{n},\;D_{n},\;G_{2},\;F_{4},\;E_{6},\;E_{7},\;\mathrm{and}\;E_{8}\right)$ and they can be used for generalized Fourier analysis of data on the fundamental region F of the corresponding Weyl groups. This theory allows for similar discretization as in the case of common Fourier discrete analysis studies, and can be used for the analysis of digitized data on the fundamental region.

Sine, cosine functions, plus $e^{\mathrm{ix}}$ are generalized to systems with nonorthonormal basis through orbit functions. Moreover, certain Weyl groups provide more types of functions, e.g., $C_{2}\;\mathrm{and}\;G_{2}$ Weyl groups allow us to define $C^{s},\;C^{l},\;S^{l}\;\mathrm{and}\;S^{s}$ functions, as described in [23]. A_2 Weyl group provide only straightforward generalization of cosine, sine and complex exponential functions. These orbit functions are generally, i.e., regardless of underlying Weyl group, defined as:

(A4) $$\Phi_{\lambda }\left(x\right)=\underset{\omega \in W}{\sum }e^{i2\pi \langle \omega \lambda ,x\rangle }$$

(A5) $$\varphi_{\lambda }\left(x\right)=\underset{\omega \in W}{\sum }\det\left(\omega \right)e^{i2\pi \langle \omega \lambda ,x\rangle }$$

(A6) $$\Xi_{\lambda }\left(x\right)=\underset{\omega \in W}{\sum }e^{i2\pi \langle \omega \lambda ,x\rangle }$$

where parameter $x\in R^{n}$ and label λ∈Q Since the orbit functions are invariant to operations of ${W\;\mathrm{or}\;W}^{e}$, respectively, we can restrict the parameter x to the fundamental region F or even fundamental region $F^{e}$, respectively. Since S-orbit function φ is anti-symmetric, it vanishes for x on boundary of F and for λ on reflection hyperplane. Through these two facts, the restriction of x and λ looks as follows:

$$\begin{array}{l} \Phi_{\lambda }\left(x\right):x\in F,\;\lambda \in P^{+},\\ \varphi_{\lambda }\left(x\right):x\in \tilde{F},\;\lambda \in P^{++},\\ \Xi_{\lambda }\left(x\right):x\in F^{e},\;\lambda \in P^{+}\cup r_{1}P^{++}, \end{array}$$

the $\tilde{F}$ denotes the interior of fundamental region F.

For the discretization of orbit functions, we choose arbitrary fixed positive integer M that defines the density of the lattice. The discrete fundamental region $F_{M}$ is constructed as an intersection of fundamental region F and stretched subset of lattice $P^{\vee }$:

(A7) FM=1MP∨Q∨∩F={s1Mω1∨+⋯+snMωn∨|s0+∑i=1ns1mi=M,s0,s1,…,sn∈Z≥0}

For discrete orbit functions we use set $\Lambda_{M}$, which is a set of discrete labels λ. The parameter M has the same meaning as for discrete fundamental region. The set $\Lambda_{M}$ is expressed as follows:

(A8) FM=PMQ∩MF∨={s1ω1+⋯+snωn|s0+∑i=1ns1mi∨=M,s0,s1,…,sn∈Z≥0}

Due to the invariance of functions to the actions of Weyl group W, and the (anti-)symmetry of functions, discrete orbit functions can be restricted in the following way:

$$\begin{array}{c} \Phi_{\lambda }\left(x\right):x\in F_{M},\lambda \in \Lambda_{M},\\ \varphi_{\lambda }\left(x\right):x\in \tilde{F_{M}},\lambda \in \tilde{\Lambda_{M}},\\ \Xi_{\lambda }\left(x\right):x\in F_{M}^{e},\lambda \in \Lambda_{M}^{e}, \end{array}$$

For further relations, scalar product over discrete fundamental region is crucial. Having two discrete functions f(x) and g(x), defined over discrete fundamental region with density M, we define their scalar product as

(A9) $${\langle f,g\rangle }_{F_{M}}=\underset{x\in F_{M}}{\sum }\varepsilon \left(x\right)f\left(x\right)\bar{g\left(x\right)}$$

Note that the region of $F_{M}$, may change depending on the used orbit function. E.g., when computing $\langle f,\varphi_{\lambda }\rangle {}_{F_{M}}$, we can omit boundary of $F_{M}$ since $\varphi_{\lambda }=0$ on the boundary of $F_{M}$, thus $\langle f,\varphi_{\lambda }\rangle {}_{F_{M}}=\langle f,\varphi_{\lambda }\rangle {}_{\tilde{F_{M}}}$.

For $\lambda ,\lambda^{\prime }\in \Lambda_{M}$, the orbit functions hold the orthogonality relation:

(A10) $$\begin{array}{c} \langle \Phi_{\lambda },\Phi_{\lambda^{\prime }}\rangle {}_{F_{M}}={\mathrm{cM}}^{2}\left|W\right|\left|{\mathrm{stab}}_{W}\left(\lambda \right)\right|\delta_{{\lambda \lambda }^{\prime }}\\ \langle \varphi_{\lambda },\varphi_{\lambda^{\prime }}\rangle {}_{\tilde{F_{M}}}={\mathrm{cM}}^{2}\left|W\right|\left|{\mathrm{stab}}_{W}\left(\lambda \right)\right|\delta_{{\lambda \lambda }^{\prime }}\\ \langle \Xi_{\lambda },\Xi_{\lambda^{\prime }}\rangle {}_{F_{M^{\prime }}^{e}}={\mathrm{cM}}^{2}\left|W^{e}\right|\left|{\mathrm{stab}}_{W^{e}}\left(\lambda \right)\right|\delta_{{\lambda \lambda }^{\prime }} \end{array}$$

M is the density of the discrete fundamental region, c denotes the determinant of Cartan matrix for the underlying group W, Cartan matrix $C={\left(c_{\mathrm{ij}}\right)}_{i,j=1}^{n},\;c_{\mathrm{ij}}=\langle \alpha_{i},\alpha_{j}\rangle$. |W| is the order of group W, ${\mathrm{stab}}_{W}\left(\lambda \right)$ is the stabilizer of the point λ under W. Generally speaking, the ${\mathrm{stab}}_{G}\left(x\right)$ is a maximum subgroup of G, such that it holds $g\left(\mathrm{zx}\right)=x\forall g\in {\mathrm{stab}}_{G}\left(x\right)$.

Since orbit functions are pairwise orthogonal over the finite region, we can expand the discrete functions f(x), g(x) and h(x) into a finite series of orbit functions:

(A11) $$\begin{array}{c} f\left(x\right)=\underset{\lambda \in \Lambda_{M}}{\sum }F_{\lambda }^{\left(\Phi \right)}\Phi_{\lambda }\left(x\right)\;x\in F_{M},\;\Phi -\mathrm{orbit}\;\mathrm{transform}\\ g\left(x\right)=\underset{\lambda \in \tilde{F_{M}}}{\sum }G_{\lambda }^{\left(\varphi \right)}\varphi_{\lambda }\left(x\right)\;x\in \tilde{F_{M}},\;\varphi -\mathrm{orbit}\;\mathrm{transform}\\ h\left(x\right)=\underset{\lambda \in F_{M}^{e}}{\sum }H_{\lambda }^{\left(\Xi \right)}\Xi_{\lambda }\left(x\right)\;x\in F_{M}^{e},\;\Xi -\mathrm{orbit}\;\mathrm{transform} \end{array}$$

Function f(x) needs to be defined on $F_{M}$, g(x) must be defined on $\tilde{F_{M}}$ and h(x) is defined on $F_{M}^{e}$. The F denotes the spectrum of discrete function f. The superscripts Φ, φ and Ξ are used for distinction between different kinds of spectra and are not commonly used.

The spectra points are given by

(A12) $$\begin{array}{c} F_{\lambda }^{\left(\Phi \right)}=\frac{\langle f,\Phi_{\lambda }\rangle {}_{F_{M}}}{\langle \Phi_{\lambda },\Phi_{\lambda }\rangle {}_{F_{M}}},\;\lambda \in \Lambda_{M}\\ G_{\lambda }^{\left(\varphi \right)}=\frac{\langle g,\varphi_{\lambda }\rangle {}_{\tilde{F_{M}}}}{\langle \varphi_{\lambda },\varphi_{\lambda }\rangle {}_{\tilde{F_{M}}}},\;\lambda \in \tilde{\Lambda_{M}}\\ H_{\lambda }^{\left(\Xi \right)}=\frac{\langle h,\Xi_{\lambda }\rangle {}_{F_{M}^{e}}}{\langle \Xi_{\lambda },\Xi_{\lambda }\rangle {}_{F_{M}^{e}}},\;\lambda \in \Lambda_{M}^{e} \end{array}$$

### A.4. Continuous Extension

One of the key properties of orbit transform is that sequence of orbit transform and inverse orbit transform preserve the processed data, e.g., $f=\sum_{\lambda \in \Lambda_{M}}\frac{\langle f,\Phi_{\lambda }\rangle {}_{F_{M}}}{{\langle \Phi_{\lambda },\Phi \rangle }_{F_{M}}}\Phi_{\lambda }$. This property is preserved even when discrete orbit function in the inverse orbit transform Equation (A11) is replaced with continuous orbit function of the same family:

(A13) $$\begin{array}{c} f\left(x\right)=\underset{\lambda \in \Lambda_{M}}{\sum }F_{\lambda }^{\left(\Phi \right)}\Phi_{\lambda }\left(z\right)\;x\in R^{n}\\ g\left(x\right)=\underset{\lambda \in \tilde{\Lambda_{M}}}{\sum }G_{\lambda }^{\left(\varphi \right)}\varphi_{\lambda }\left(z\right)\;x\in R^{n}\\ h\left(x\right)=\underset{\lambda \in \Lambda_{M}^{e}}{\sum }H_{\lambda }^{\left(\Xi \right)}\Xi_{\lambda }\left(z\right)\;x\in R^{n} \end{array}$$

In this case, we obtain a continuous extension of the original data. As proven, see [2], certain families of orbit functions can provide high-quality approximation with quick convergence to the original continuous data.
